# The link between corporate sustainability and willingness to invest: new evidence from the field of ethical investments

**DOI:** 10.1007/s00187-022-00340-z

**Published:** 2022-05-30

**Authors:** Volker Lingnau, Florian Fuchs, Florian Beham

**Affiliations:** University of Kaiserslautern, Kaiserslautern, Germany

**Keywords:** Sustainability management, Business ethics, Ethical investment, Empirical study, Factorial survey, Willingness to invest

## Abstract

In recent decades, academia has addressed a wide range of research topics in the field of ethical decision-making. Besides a great amount of research on ethical consumption, also the domain of ethical investments increasingly moves in the focus of scholars. While in this area most research focuses on whether socially or environmentally sustainable businesses outperform traditional investments financially or investigates the character traits as well as other socio-demographic factors of ethical investors, the impact of sustainable corporate conduct on the investment intentions of private investors still requires further research. Hence, we conducted two studies to shed more light on this highly relevant topic. After discussing the current state of research, in our first empirical study, we explore whether besides the traditional triad of risk, return, and liquidity, also sustainability exerts a significant impact on the willingness to invest. As hypothesized, we find that sustainability shows a clear and decisive impact in addition to the traditional factors. In a consecutive study, we investigate deeper into the sustainability-willingness to invest link. Here, our results show that improved sustainability might not pay off in terms of investment attractiveness, however and conversely, it certainly harms to conduct business in a non-sustainable manner, which cannot even be compensated by an increased return.

## Introduction

Over the past decade, the issue whether it might “pay to be good” has brought up a diverse set of research approaches and perspectives on the impact of sustainability commitments for business success (e.g., Al-Hadi et al., 2017; Eccles et al., 2014; Wang et al., 2016; Yu & Zheng, 2020). While several studies explore the interrelationship between ethical consumption preferences and consumers’ willingness to pay (e.g., Andorfer & Liebe, 2012; Li & Kallas, 2021; Tully & Winer, 2014), measuring the demand side of production of different products and services like coffee (e.g., Andorfer & Liebe, 2015; Lingnau et al., 2019), chocolate (e.g., Didier & Lucie, 2008; Poelmans & Rousseau, 2016), and renewable energy (e.g., Soon & Ahmad, 2015), some other studies have been focusing on the supply side of business, and in specific the issue whether sustainability might be attractive from an investor’s perspective (e.g., Barber et al., 2021; Revelli & Viviani, 2015).

Traditionally, the standard-economic theory assumes that investment decisions are solely influenced by three factors: expected returns, involved risks, and liquidity preferences of the investor (Becker, 2010; Eichhorn & Towers, 2018; Schmeisser et al., 2011). Depending on the preferences of the investor, these factors are weighted differently. In this context, return, risk, and liquidity are not independent of each other but must be viewed in a reciprocal interplay. A high return often goes hand in hand with a high risk or long investment period, while investments with short maturities and low risk promise relatively low returns. Because of this interdependence in terms of return, risk, and liquidity, the relation is often referred to as the “magic triangle” of investment. However, such traditional economic thoughts face their limitations in the context of increasing sustainability demands, which are not reflected by these considerations but increasingly enforced by stakeholders and in specific by some shareholders as well. Beal et al. (2005, p. 66) summarize this expansion in relevant decision parameters, emphasizing: “One particular type of behavior that has emerged over the last 20 years or so is the desire to invest ethically”. Similarly, Revelli and Viviani (2015, p. 158) state that “in the past 20 years, socially responsible investing (SRI), which embodies ethical values, environmental protection, improved social conditions and good governance, has increasingly attracted the interest of individual and private investors, as well as academics”. Looking at these changes from a conceptual perspective, thus, the traditional “magic triangle” is extended by a fourth dimension of sustainability and becomes a “magic square” (Von Wallis & Klein, 2015). In this case, private investors do not base their decisions exclusively on financial factors but rather seek investments that are in line with their personal values (Pasewark & Riley, 2010).

The issue of increasingly demanded sustainability concerns is likewise more and more reflected by research in Management Accounting (see Soderstrom et al., 2017) as one major task of Management Accounting is providing management with useful information for decision-making via performance figures, which also can be utilized for reporting to stakeholders, and in particular, shareholders. Therefore, if research could empirically substantiate that sustainability issues are increasingly gaining momentum as decisive success factors for businesses, Management Accounting would be challenged by delivering more indicators representing, for instance, the social and environmental performance and therefore complementing the traditionally applied, economically-based indicators. In addition, the amplified relevance of sustainability performance could lead to a redesign of traditional incentive schemes to include a stronger emphasis on sustainability orientation. In such vein, also Soderstrom et al. (2017, p. 60) conclude that “there are increasing pressures from investors and other stakeholders for firms to consider sustainability-related factors within their management control systems”.

Observing the current state of research, although traditionally there has been a strong focus on the impact of social or environmental responsibility for business performance (for an overview see Revelli & Viviani, 2015), newer research has also been increasingly come to investigate the individual investment decisions in the domain of SRI. As shareholders are (amongst others) one of the major, or “primary”, stakeholders (Clarkson, 1995; Freeman, 2010) of an enterprise, a deeper understanding of factors influencing shareholders’ willingness to invest (WTI) is of particular relevance. Yet, most studies that already explored the attractiveness of SRI have remained largely theoretical, utility oriented or if empirically based, been focusing predominantly on the motives or socio-demographic factors of sustainability-oriented investors. In particular, more experimentally-based settings remain relatively scarce (Soderstrom et al., 2017). However, an experimental approach, as represented in traditional laboratory settings, but also embedded in the so-called vignette approach (see following discussion), would be of great relevance as we first need to obtain a better understanding of sustainability as an *overall* decision parameter in the WTI context. Specifically, up-to-date, to our knowledge there is no research conducted in an integrated design which allows to observe the impact of sustainability in comparison to the traditional decision parameters, i.e., return, risk, and liquidity. In addition, there are currently to our knowledge no studies that explore whether there is a difference between the potentially positive impact of additional sustainability measures and, conversely, the possibly negative impact of bad sustainability practices on WTI.

Given these considerations and open research questions, our paper contributes to present research in two major ways, represented in two empirical studies. After providing a review over the current state of research, our first study will investigate the relevance of sustainability as a major investment parameter, controlling for several socio-demographics. In addition to already existing literature, we will simultaneously explicitly integrate the traditional investment parameters (return, risk, liquidity), which allows for a first-time joined evaluation of impact on an investment decision. In this context and with our integrated model, we can show that sustainability is, besides return, risk, and liquidity, indeed a very strong decision parameter. Our second study will then deeper investigate into the potential difference between an amplified sustainable conduct and a lack of sustainability commitment from an investor’s perspective. In this context, we can show that an additional, above-average commitment to sustainability does not increase WTI, however, a lack of sustainability clearly leads to a significantly reduced WTI, which cannot be compensated even by an increased return prospect.

## Theoretical background and previous studies

While the relationship between Corporate Social Performance (CSP) and Corporate Financial Performance (CFP) is intensively discussed in research, with most reviewing studies finding a positive link (Eccles et al., 2014; Orlitzky et al., 2003; Van Beurden & Gössling, 2008; Wang et al., 2016), the debate about corporate responsible behavior also shifted to the question whether sustainable stocks, funds, indices, and other ethical investment opportunities are more profitable from an investor’s perspective. From this point of view, investors acknowledge that socially and environmentally sustainable corporate behavior may not only increase financial corporate performance but also can have a crucial value for investors in itself (Cheney, 2004; Hart & Milstein, 2003). In this context, especially SRI has become a preferred subject in academia because it has opened a possibility of examining not solely financially driven aspects of investment decisions but also non-financial issues. Similarly, Hellsten and Mallin (2006, p. 395) summarize these considerations: “While owners and investors care about […] their profits, they increasingly also care about other social issues that their investments may one way or another influence”.

Although SRI is often linked to the concept of Corporate Social Responsibility (CSR) due to its focus on ethical and sustainable aspects, both concepts are not the same. For instance, Sparkes (2002, p. 42) emphasizes: “CSR and SR investing are in essence mirror images of each other. […] CSR looks at this from the viewpoint of companies, SR investment from the viewpoint of investors in those companies”. Like the ambiguity in conceptual interpretation of the term CSR, which is in the following shortly defined by analogy to the three pillars of sustainability as integrating social and environmental topics into corporate (governance) actions (Pinner, 2003), until today, there exists no common definition of the term SRI (Cowton, 1994; Louche, 2004). In such vein, also Derwall et al. (2011, p. 2137) state: “Socially responsible investing (SRI) has undergone tremendous development since it emerged as a faith-based initiative in the eighteenth century”. SRI in its current form is often claimed to have emerged in the US during the 70s and early 80s as a consequence of concern for non-economic motives and became global practice by the early 2000s (Sparkes, 2002) sometimes focusing more on ethical and sometimes more on financial aspects. While Cowton (1999) summarizes the discussion on the different investment types as “matter of taste”, Sandberg et al. (2009, p. 521) find that definitions of SRI are overwhelmingly consistent in the interpretation that they refer to an “integration of certain non-financial concerns, such as ethical, social or environmental, into the investment process”. Although most often the concept of SRI is defined in such broader^1^ terms, in the following article, we restrict ourselves to an enquiry of the social and environmental dimension. Hence, we consider SRI in a narrower, CSR-oriented interpretation, as an investment process that integrates investors’ concerns on social and environmental issues.

Having a closer look on SR investors, “SRI is comprised of an investment community encompassing a wide range of individuals and groups (including religious groups, universities, and some pension and mutual funds) interested in criteria other than simple return on investment” (Waddock, 2003, p. 369). In this context, also Cohen et al. (2011, 2015) as well as Reimsbach et al. (2018) show that investment decisions of professionals and non-professionals vary regarding their preferences and reliance on non-financial information (e.g., professionals require information which is more detailed, comprehensive, as well as credible). In contrast to some SRI literature that focuses solely on institutional investors or other investment groups, the following article refers to private, primarily non-professional investors as a specific investment group that is rather influenced by consumer motives than being “cooked up by Wall Street” (Von Wallis & Klein, 2015, p. 64).

SRI decision-making is sometimes further linked to the field of ethical consumption, indicating a positive relationship between corporate CSR commitments and consumers’ willingness to pay for products and services produced in an ethical way as the two meta-analyses by Andorfer and Liebe (2012) as well as by Tully and Winer (2014) show. Comparable to the field of ethical consumption, showing that consumers are driven by different motives and concerns, also SR investors can derive personal satisfaction in different ways (Pasewark & Riley, 2010). For instance, Bollen (2007) argues that an investor’s utility function is not solely based on the standard risk-reward optimization but also can include personal and societal values. Therefore, SRI can mean different things to different investors. The paradox of socially responsible investing is summarized by Gasparino and Tam (1998) stating that “one person’s taboo is another person’s sacred cow” (also see Von Wallis & Klein, 2015, p. 67). Therefore, and in contrast to existing assumptions that treat SR investors as a homogeneous group, private investors are in reality likely to be heterogeneous in their investment preferences. For instance, with regard to the group of shareholders, Lingnau and Fuchs (2018) show concisely that the conjecture of stockholders as a homogenous group that solely wants to maximize the financial return is only appropriate under the highly unrealistic assumption of perfect markets. In line with this reasoning that not every investor is driven by purely financial return maximization motives, Andreoni and Miller (2002) identify that only one quarter of the investigated population are pure money-maximizers, which implies that three quarters are willing to give up income for non-pecuniary utility. Bauer and Smeets (2015) support this view and show empirically for a segment of socially responsible banking clients that they also gain non-pecuniary benefits from investing in socially responsible investments. Following these considerations and empirical results, it seems reasonable to assume that personal investment decisions are primarily linked with individual values and motives, which can be driven by economic motives but also by ethical objectives. For instance, ethical motives can be the “feel good” effects from social investing (Michelson et al., 2004; Schueth, 2003; Webley et al., 2001), sociocultural influences on perceptions of corporate responsibility (Hofstede & Hofstede, 2005; Katz et al., 2001), or even religious views (Brammer et al., 2007; Naber, 2001). With regard to cultural and religious values, Sjöström (2011, p. 9) summarizes that the interpretation of SRI and the portfolio composition can even vary among different cultures: “A Spanish SRI fund may be defined different to an Australian SRI fund, a Shariah fund may include different investment criteria than an environmental fund, and so on” (also see Trinks & Scholtens, 2017).

As previous results in research have revealed, there exists some empirical evidence indicating that ethical values are relevant in the motives and outcomes of SR investors’ decision-making (Schueth, 2003; Sparkes & Cowton, 2004). Hummels and Timmer (2004) support this view by measuring private investors’ motives on a continuum that ranges from a strictly ethical orientation to a strictly financial orientation. Examining 563 SR investors, Nilsson (2009) finally distinguishes three different types of SR investors. The first type of SR investor values financial return over social responsibility, the second type values social responsibility over financial return, and the third type of investor is not predominately driven by financial or non-financial aspects, valuing both economic return and social responsibility. With regard to the first and second investor type, Derwall et al. (2011) are even able to find differences in investment decisions. While ethical value-driven investors primarily use “negative” screens to avoid controversial stocks, the profit-driven segment uses “positive” screens. Finally, also Vyvyan et al. (2007) identify significant differences in investment attitudes regarding SRI criteria between environmentalists and non-environmentalists, however, no difference could be detected in terms of investment selection by surveying 318 people in Australia.

In addition to an examination of private investors’ personal motives, some of the SRI literature has been investigating what kind of socio-demographic factors (gender, age, income, education, social network) influence personal investment decisions (e.g., Ostrovsky-Berman & Litwin, 2018). For example, Rosen et al. (1991) conducted a study surveying 4000 individual investors in SRI funds and revealed that SR investors are younger and better educated. The results of Rosen et al. (1991) are also supported by a number of other studies showing that SR investors tend to be younger (e.g., Diamantopoulos et al., 2003; Hayes, 2001; Laroche et al., 2002). Focusing on further socio-demographic aspects, Sparkes (2002) shows that SR investors are well-educated and have a higher income. In this context, Tippet (2001) as well as Vinning and Ebreo (1990) show that SR investors are generally wealthier than their conventional counterparts, stating that they may be more willing to tolerate an “ethical penalty” (Williams, 2007). Classifying SR investors regarding their gender, Tippet (2001) as well as Tippet and Leung (2001) additionally find that SRI is predominately linked to female rather than male investors. These results are also in line with the findings of Schueth (2003), who suggests that SRI in the US is driven by general improvements in education levels and from the wider involvement of women in the equities market. Examining the gender of SR investors, also Diamantopoulos et al. (2003) and Laroche et al. (2002) find in their studies that SR investments tend to be linked to women. With a questionnaire of 2464 SRIs from 20 countries, also Cheah et al. (2011) reveal that younger and female SR investors regard social and environmental aspects as important. These results are also in line with Dorfleitner and Nguyen (2016) who emphasize that female and younger investors seek to invest a higher proportion in socially responsible investments. However, reviewing the influence of socio-demographic factors in the SRI literature, the results are not consistent (Siddiqui, 2018). For instance, and in contrast to the results aforementioned, examining the investment behavior of 1000 investors, Lewis and Mackenzie (2000a) find that SRIs are not a single phenomenon of younger and wealthier investors but can be linked to middle-aged people with average income as well. The results of Lewis and Mackenzie (2000a) are supported by McLachlan and Gardner (2004), Williams (2007), and Berry and Yeung (2013) that also found no evidence of significant differences for SRI regarding socio-demographic factors like e.g., gender, educational level, income, and age.

Similar to the connection of CSP and CFP, the research results of SRI and stock market performance are ambiguous and sometimes even contradictory. Consequently, they are intensively discussed in research (Chegut et al., 2011). While some researchers identify a positive impact of SRI on investment performance, other authors find no significant difference or even a negative impact of SRI in comparison to conventional investments. Reviewing existing studies in the field of SRI, for example Revelli and Viviani (2015) show in a meta-analysis of 85 studies and 190 experiments that the consideration of corporate social responsibility in stock market portfolios do neither indicate a weakness nor a strength compared to their conventional counterparts. These results are also supported in a more current meta-analytic review by Kim (2017) examining the investment performance based on 205 US samples showing that the financial return of SRI is not significantly different from conventional investments. Independent of the before mentioned meta-analyses in SRI, there are basically three different arguments regarding the effect of SRI on investment performance emphasizing a positive, negative, and no impact on stock and portfolio performance (Hamilton et al., 1993; Revelli & Viviani, 2015; Von Wallis & Klein, 2015).

A plausible argument that SRIs outperform conventional investments can be found in the theoretical discussion that corporations with a high ethical standard can have a source of competitive advantage (Porter & Kramer, 2011) and thus can increase an investor’s return. Another explanation that SRIs perform better than conventional investments is based on stakeholder theory (Freeman, 2010) stating that taking into account the expectations of different interest groups (stakeholders) and improving social and environmental aspects creates value for the business and thus can exert a positive impact on stock performance. This argumentation is also in line with research on social preferences stating that positive acts are reciprocated by positive responses, while negative ones are sanctioned by negative behavior in return (e.g., Fehr & Schmidt, 2006). Another argument that is also supported by empirical evidence is that socially responsible corporations have greater access to financial resources, which reduces their cost of equity (Heinkel et al., 2001; Mackey et al., 2007; Merton, 1987), increases demand and raises the prices of SRI stocks. In this context also Hong and Kacperczyk (2009) discover that so called “sin” (screening out)^2^ companies, i.e., publicly traded companies involved in the production of alcohol, tobacco, and gambling are punished by capital markets, due to the higher cost of capital. Finally, also Bauer et al. (2005, 2006) reveal that the performance of SRI funds improve over time, which can be interpreted as a learning effect. In accordance to the argumentation of Bauer et al. (2005, 2006) also Cummings (2000) as well as Barnett and Salomon (2006) identify that the SRI funds’ performance is better in a long-term evaluation and especially with regard to crises like for example the COVID-19 pandemic (e.g., Liu, 2020). A similar picture can be shown in the long term for high sustainability companies regarding stock market as well as accounting performance (Eccles et al., 2014).

In contrast, a conceptual argument that supports the argumentation that SRI lowers equity performance is based on portfolio theory (Markowitz, 1952). Through the lens of portfolio theory, the SRI selection and exclusion of certain stocks reduces investment opportunities and consequently investors’ ability to diversify their portfolio. In this context, also Rudd (1981) argues conceptually that each time a portfolio is constrained, its performance suffers. Following the argumentation of Rudd (1981), also Clow (1999) remarks that SRI is linked with a sector bias restricting the number of investment areas and consequently can increase market risk. Besides the before-mentioned arguments, SRIs are also linked with higher diversification costs in business literature (Girard et al., 2007). The increasing diversification costs can arise on one hand from higher costs in gathering and interpreting information and on the other hand at least by determining which stock belongs to the SRI universe (Revelli & Viviani, 2015). In this context, also Bauer concludes that SRI lowers economies of scale, which leads to higher transaction costs and management fees (Barnett & Salomon, 2006; Bauer et al., 2005). Besides these mainly theoretical arguments, there exist also empirical results revealing that conventional assets perform better than their ethical counterparts. For example, Renneboog et al. (2008b) show that SRI funds of France, Ireland, Sweden, and Japan perform significantly worse than conventional funds by 4–7% per annum during the period of 1991–2003. In line with the result of Renneboog et al. (2008b), also Fabozzi et al. (2008) and Statman and Glushkov (2009) discover that sin stocks perform better than other stocks.

While some theoretical as well as empirical reasons indicate a positive or a negative impact of SRI, some other arguments pinpoint to the conclusion that there is no difference between SRI and conventional investments concerning financial performance (Revelli & Viviani, 2015). A first theoretical argument for such view can be found in the efficient market hypothesis. For example, Hamilton et al. (1993) state that in a world with semi-efficient markets, social responsibility is not priced in the market. As a consequence, SR investors who want to sell their shares find enough conventional buyers for them, so share pricing is not affected (see also Hamilton et al., 1993, p. 63; Von Wallis & Klein, 2015, p. 74). Besides this rather theoretically driven argument, another reason might arise nowadays due to stricter legal and ethical requirements such that SR stocks, funds, and indices do not differ from their conventional counterparts as much as one would expect. Supporting these theoretical considerations, for instance, Schröder (2004) empirically found no significant underperformance comparing US, German, and Swiss SRI funds with their specific benchmarks. Correspondingly, Bauer et al. (2005) explored German, UK, and US ethical mutual funds and found no evidence for significant differences in risk-adjusted returns between ethical and conventional funds during the period of 1990 and 2001. This result is consistent with Hamilton et al. (1993), Reyes and Grieb (1998), Goldreyer and Diltz (1999), Statman (2000), Bello (2005), and Utz and Wimmer (2014), who did not find significant differences in the performance of SR and conventional funds, even though they examined different samples, time periods and countries.

Summarizing the review on the existing array of research in the field of SR investments, it becomes evident that previous studies have been predominantly focused on three aspects. First, some studies have been concerned with whether sustainability issues increase business performance economically. Second, some of the existing studies have been investigating whether it pays off from an investor’s perspective to invest into sustainable stocks, funds, indices, and other investment opportunities. Third, several studies have been exploring the motives to invest into sustainable businesses, specifically with a focus on the character traits and socio-demographics of ethical investors. However, in present research one major aspect has received particularly less consideration: the impact of sustainability issues on an individual’s *willingness to invest* into a company’s stock. As sufficient equity provision is of prime relevance for business success, such perspective is of particular importance. Hence, it would be highly relevant to better understand the impact of sustainability issues in an investment decision, especially in comparison to the traditional triad of risk, return, and liquidity. Therefore, and to examine this issue in greater detail, at first, it is relevant to explore whether the transition from the traditional triad to an advanced model, also explicitly covering the sustainability dimension, can be empirically justified and if so, how much the respective impact will appear to be in comparison. This subject is explored by the following first study.

## Study 1

### Empirical hypotheses

Based on the previous considerations from standard-economic theory, the traditional factors influencing an investment decision are based on the triad of return, risk, and liquidity, where greater return and liquidity are always favored, while conversely less risk is preferred, usually measured by variance in return (Becker, 2010; Eichhorn & Towers, 2018; Schmeisser et al., 2011). However, as previously discussed, a conceptual restriction to these traditional three parameters clearly faces its limits in the increasing stakeholder demands for sustainability – also, and particularly, on the side of shareholders. These changes are markedly reflected by research discovering sustainability as a major construct for understanding investment decisions (Ambec & Lanoie, 2008; Rhodes, 2010), which is equally mirrored in the conceptual change from a “magic triangle” to a “magic square” (Von Wallis & Klein, 2015).

Such an extension to four crucial dimensions in an investment decision can be substantiated with theorizing from the domain of social and ethical preferences research, thus, transcending a motivationally narrow standard-economic argumentation. Traditionally, orthodox economics’ theorizing is based on the concept of *homo oeconomicus*, who is traditionally perceived as being solely motivated by an accumulation of material wealth and in principle indifferent toward other individuals or motives, except if these are perceived as relevant for the former, i.e., personal enrichment (Boulding, 1969; Homans, 1961). In opposition to that, several streams of research have criticized these assumptions on theoretical as well as empirical grounds. From an ethical point of view, already Sen (1977, p. 336) famously labeled the homo oeconomicus with these exclusively materially, self-oriented preferences a “rational fool” and “social moron” leading Sen in turn to propose the concept of *meta-preferences* representing ethical concerns coming into place when normative issues are affected. With regard to the sustainability impact on WTI, one could correspondingly assume that economic considerations are still relevant but complemented by genuine sustainability concerns that could strongly affect the final investment decision. These conceptual considerations are also empirically supported, especially by research on social and in particular sustainability preferences. In this area, there is a great amount of research showing that individuals possess economic but also several social motives (e.g., Fehr & Gächter, 2002; Fehr & Schmidt, 2006; Fehr et al., 2005). One of the strongest and oldest motives in social preferences is the tendency to reciprocate, stating that behavior being perceived as benevolent is answered by friendly acts, while conversely negative behavior, led by selfish or hostile intentions, is punished (e.g., Bolton & Ockenfels, 2000; Falk & Fischbacher, 2006; Fehr & Gächter, 2000). Concerning such evaluations, an increasing amount of research has covered sustainability preferences, particularly in the context of ethical consumption, but also increasingly in the area of investments (e.g., Hoang & Phang, 2020; Kim, 2017; Lins et al., 2017; Phang & Hoang, 2021; Revelli & Viviani, 2015). Connecting these lines of thought, it seems plausible to assume that individuals are increasingly valuating and rewarding additional corporate engagement that transcends traditional economic performance indicators. Therefore, being a responsible business unit should (ceteris paribus) lead to a favorable intention with an on average greater WTI on the side of potential investors. In this context, our following studies will particularly focus on the investment decisions of private investors as these are more and more active in the domain of investment, specifically with the increasing availability of trading platforms. Summarizing these considerations, we state our first research hypothesis as follows:

- **H1** Corporate sustainability exerts a positive influence on the willingness to invest for private investors.

### Methods, procedures and sample

To investigate the hypothesis previously elaborated, we applied a factorial survey approach. In general, the method “integrate[s] elements of survey research and classical experiments” (Oll et al., 2018, p. 26). By doing so, it enables the researcher to combine some of the advantages of traditional laboratory experiments, like the controlled impact of confounding variables and greater internal validity, with the advantages of traditional survey studies that usually permit a larger number of participants, thus yielding an augmented statistical power and increased significance of results. Although the approach might not “completely resolve the problems caused by the difficulty of conducting true experiments with representative samples”, it can “by combining some of the advantages of experimental and survey designs, […] provide stronger tests of causal hypotheses than other surveys and more generalizable findings than experiments” (Schutt, 2018, p. 244). According to the classic definition by Alexander and Becker (1978, p. 94), vignettes, as the major concept behind the factorial survey approach, can be understood as “short descriptions of a person or a social situation which contain precise references to what are thought to be the most important factors in the decision-making or judgment-making processes of respondents”. Likewise, several newer definitions fundamentally rely on such conceptualization. For instance, Atzmüller and Steiner (2010, p. 128) refer to a vignette as “a short, carefully constructed description of a person, object, or situation, representing a systematic combination of characteristics”, while Auspurg and Jäckle (2017, p. 490) define a vignette as a description of “a hypothetical situation or object as having various attributes (dimensions)”. The major idea of the approach is now to consciously and systematically vary some elements in the situational descriptions (vignette factors), constituting an equivalent to the traditionally controlled experimental stimulus. The effects of such variation are then observed using traditional survey designs in which the participants’ responses are gathered. Besides the widespread denomination as “factorial survey (experiment)” (e.g., Auspurg & Hinz, 2015; Oll et al., 2018) the approach is sometimes also referred to as “vignette studies” (e.g., Atzmüller & Steiner, 2010; Martin, 2012) or “vignette analysis” (e.g., Dülmer, 2007). The vignette approach can be applied to a diverse set of domains and is “well suited to dealing with the complex interplay of societal-, organizational- and individual-level factors […] and to studying the principles underlying human perceptions, attitudes, values, social norms, and (anticipated) behavior” (Oll et al., 2018, p. 26).

To explore the empirical hypothesis on the impact of sustainability on WTI, we designed sixteen vignettes, comprising the three traditional decision parameters (return, risk, and liquidity), which were complemented by the factor of sustainability, each with two possible characteristics (good/bad outcome) (for detailed information see Appendix 1). Before the presentation of detailed descriptions, the participants always received general information to ensure a comparable level of basic investment knowledge. Then, the participants were asked to put themselves in the scenario of having inherited 10,000 €, from which a certain amount could be invested into stocks. Subsequently, the specific details concerning the four decision parameters were presented according to the randomly assigned vignette. After presentation of the respective vignette, we asked the participants to indicate the amount of money they were willing to invest in that given situation via a slider that enabled the individuals to choose a value between 0 and 10,000 €, also providing a click-box explicitly indicating that the individual would not invest into the company at all (0 €).

The vignettes themselves were extensively pre-tested with several student and non-student subjects not participating in the study to ensure that the vignettes were easy to understand and precise. Concerning the design of distribution, several possibilities exist. The question of which design to apply in the context of vignette distribution is intensively discussed in research. Principally, one can distinguish three methods of dissemination: The within-subjects design, distributing the entire vignette universe (or at least the same sample) to all participants, the between-subjects design, providing each participant only with one vignette and the mixed design, which combines both approaches (Atzmüller & Steiner, 2010). For our empirical study, we chose the *between-subjects design*, i.e., we provided exactly one vignette per participant. Although such design increases the number of participants required to obtain significant results, for the research at hand, the approach seems superior as it helps avoiding statistical distortions through guessing the research design as well as order or fatigue effects. In such manner, also Charness et al. (2012, p. 4) emphasize that if “the goal should be to achieve an independent evaluation of each scenario by participants […] a between approach may be preferable”.

To ensure that our results would not solely be significant but also possess sufficient statistical power, we conducted an ex-ante power analysis. Assuming a for regression analyses medium effect size of Cohen’s f^2^ = 0.15,^3^ we obtained a minimum amount of 135 participants. In our first study, in total 512 individuals (314 male, 192 female, else/unspecified: 6) participated, completing the survey with a rather equal distribution of around 32 participants per vignette. The participants did not significantly differ between the vignette conditions regarding socio-demographics or personality traits. Most participants were university students (57.2%), while also several employees participated (31.3%, else: 11.5%). The average age in the first sample was 27.3 (SD = 7.2) years with a minimum of 16 and a maximum of 68.

### Results

Observing our empirical results, already a simple descriptive analysis with subsequent t-tests (one-tailed) pinpoints to the conclusion that not only the traditionally expected factors of return, risk, and liquidity affect the WTI but equally the hypothesized dimension of business sustainability. In such manner, first, in the *low return* group, the individuals indicated an on average WTI of 2439.06 €, while given a *high return* expectation, the stated WTI increases to 3075.00 € (∆ = 635.94; *p* = .003). Second, concerning the impact of risk, as expected in the *high risk* condition, individuals stated an average WTI of 2225.39 € which increases to 3288.67 € in the case of *low risk* (∆ = 1063.28; *p* < .001). Third, also the results of the factor liquidity show the traditionally assumed effect direction. While given the information on *low liquidity* the on average stated WTI is 2454.09 €, in the case of *high liquidity* such WTI increases to 3062.35 € (∆ = 608.26; *p* = .004). Finally, and as a first indication supporting our hypothesis, also sustainability shows a quite strong impact between the vignettes. Hence, in the case of *bad sustainability* practices the on average stated WTI is merely 2017.19 €, while in the *good sustainability* condition, the WTI increases to 3496.88 € (∆ = 1479.69; *p* < .001).

To further investigate the outlined empirical hypothesis, we conducted a multiple OLS regression analysis containing the traditional three parameters of return, liquidity, and risk, further including the hypothesized factor of corporate sustainability as well as several control variables to reduce omitted variable bias. As controls, we included age, gender, and income along with the status of being a student to check whether the in-part student surrogation would affect responses. Furthermore, we also controlled for the investment experience (5-point scale) as more experienced investors might deviate in their responses from relative novices. In addition, since the average investment sum per year could influence the specified WTI, such measure was also included as an ordinal scale. In this context, the return preference was reflected by the self-stated satisfying return expectations in general, while risk preferences were estimated applying the scale by Weber et al. (2002). Finally, we also included a measure for liquidity preferences (7-point scale), while sustainability preferences were gathered using the scale by Balderjahn et al. (2013).

Running the regression analysis, we find that all four decision factors demonstrate a highly significant impact on WTI with the traditionally assumed or hypothesized sign direction. Thus, at first, our study underpins the traditional assumptions that return and liquidity exert a positive impact on WTI, while for risk, the association is negative. In addition to that and referring to H1, sustainability shows a significant, positive impact, which is interestingly even the strongest, followed by risk, return, and liquidity. Hence, H1 can be clearly supported by our empirical data leading us to conclude that the expansion of the traditional “magic triangle” to the “magic square” is empirically substantiated. Regarding the included controls, most variables do not exert a significant effect. Particularly the non-significance of the status of being a student is reassuring as therefore the in-part surrogation seems not to have led to a significant response deviation. Interestingly, likewise the investment experience did not influence the stated willingness to invest, however, the average investment sum showed a significant, positive impact. Finally, also an increasing risk preference generally leads to a larger WTI (see Table 1).

**Table 1:** OLS regression results, Study 1

	Beta	SE	t	Sig
(Constant)	− 1951.969	979.408	− 1.993	.047
Return	709.578	220.604	3.217	.001***
Risk	− 995.321	223.347	− 4.456	.000***
Liquidity	603.641	219.781	2.747	.006***
Sustainability	1463.631	220.309	6.644	.000***
*Control variables*				
Age	13.045	17.871	.730	.466
Gender	− 51.419	246.550	− .209	.835
Income	79.221	63.888	1.240	.216
Student	20.353	279.163	.073	.942
Investment experience	− 41.994	121.503	− .346	.730
Average investment sum	207.320	95.796	2.164	.031**
Return expectations	− 2.892	1.822	− 1.587	.113
Risk preference	110.156	20.451	5.386	.000***
Liquidity preference	66.404	88.644	.749	.454
Sustainability preference	.699	1.308	.535	.593

R^2^ = .223. N = 452, 60 participants omitted as they did not fully answer to all items. Gender coding: male = 0, female = 1. Beta: Unstandardized regression coefficients. Dependent variable: Willingness to Invest

**p < .05; ***p < .01

Having established the general link between corporate sustainability and an investor’s willingness to invest, it seems vital to further investigate this relationship. Thus, in a subsequent study, we will particularly focus on the question whether positive and negative sustainability issues are treated alike or if these are evaluated differently.

## Study 2

### Empirical hypotheses

As previously discussed, besides the traditional return, risk, and liquidity scheme, also corporate sustainability commitments are increasingly considered as having a significant influence on decision-making processes, which should equally hold in the area of investments, i.e., concerning an individual’s willingness to invest. For subsequently deriving the hypotheses of Study 2, we draw once more on the considerations outlined in the context of the first study. In particular, we refer again to the extant research on sustainability preferences, which have been particularly investigated in the domain of ethical consumption but which should also hold as a general motive in the domain of ethical investments (e.g., Hoang & Phang, 2020; Kim, 2017; Lins et al., 2017; Phang & Hoang, 2021; Revelli & Viviani, 2015). This basic motive is then connected to the domain of social preferences and in particular to the motive of reciprocity (e.g., Bolton & Ockenfels, 2000; Falk & Fischbacher, 2006; Fehr & Gächter, 2000), indicating that good (i.e., sustainable) corporate acts should be rewarded (e.g., Al-Hadi et al., 2017; Lins et al., 2017), while actions perceived as bad or wrong should be punished by reduced willingness to invest. In such vein, different studies show that “positive CSR performance can reduce the degree of financial distress […] and […] minimise the effects of adverse events on both the stock and bond prices” (Phang & Hoang, 2021, p. 2), while such positive effects are reduced or even inverted when corporations are under suspicion of greenwashing (Lyon & Maxwell, 2011). Observing again the current state of research, it becomes evident that most studies existing only have explored social, environmental, or ethical aspects in general (e.g., Hudson, 2005; Lewis & Mackenzie, 2000a). However, from a business perspective, sustainability aspects can be expressed in social as well as environmental terms (Dyllick & Hockerts, 2002; Epstein & Roy, 2003; Schaltegger & Wagner, 2017). Hence, in the context of the second study, we will reflect these considerations by explicitly differentiating between social and environmental sustainability aspects. Therefore, starting with social sustainability, we set forth the following hypothesis:

- **H2a** Private investors are willing to invest more into shares of socially sustainable companies than into companies whose sustainability is not known (control group).

And correspondingly, for the dimension of environmental sustainability:

- **H2b** Private investors are willing to invest more into shares of environmentally sustainable companies than into companies whose sustainability is not known (control group).

Such a link should particularly hold for combining those commitments, leading us to hypothesize:

- **H2c** Private investors are willing to invest more into shares of environmentally and socially sustainable companies than into companies whose sustainability is not known (control group).

In addition, it would be relevant to explore whether combining social and environmental commitments may lead to an even greater improvement in willingness to invest than an increase in only one sustainability dimension. These considerations can be based on previous theorizing: If both social as well as environmental sustainability increases WTI in comparison to the control group, then taken together, both effects combined should be greater than the effect by an improvement in only one sustainability dimension. From a theoretical point of view such an aggregation effect could be theorized on grounds of an increasing plausibility, consistency and schematic “fit” (Axelrod, 1973) of corporate sustainability signals with true intentions. I.e., an in the eyes of potential shareholders greater self-binding corporate commitment should lead to a further increase in WTI compared to solely increasing the sustainability performance in one dimension. Accordingly, this leads us to the following pair of hypotheses:

- **H3a** Private investors are willing to invest more into shares of environmentally and socially sustainable companies than into solely socially sustainable companies.
- **H3b** Private investors are willing to invest more into shares of environmentally and socially sustainable companies than into solely environmentally sustainable companies.

Furthermore, and in contrast to the theorized impact of positive sustainability related commitments, it is equally important to investigate whether an explicit lack of sustainability might decrease WTI. This is particularly relevant as for instance in the domain of sustainable consumption the business related consequences of negative sustainability have received increasing attention in empirical research approaches (e.g., Lingnau et al., 2019; Moosmayer, 2012; Trudel & Cotte, 2009). Yet, for the field of sustainable investments there is a clear lack of studies explicitly investigating not only the positive effects of increasing sustainability commitments but equally the potentially negative business effects of a perceived lack in sustainability efforts. This seems particularly remarkable as already the survey-based study by Lewis and Mackenzie (2000b) found that 83.9% of ethical investors stated a clear desire to avoid harmful companies. Based on these thoughts, we consider it particularly relevant to cover the potential harm to business, as measured by reduced WTI, following an apparent lack of sustainability commitment. Hence, we set forth the following hypothesis:

- **H4** Private investors are less willing to invest into shares of non-sustainable companies than into companies whose sustainability is not known (control group).

In addition, as several findings from psychology demonstrate, individuals differ in their evaluation of positive and negative events. Usually, participants show a much stronger reaction toward negative than toward positive information. In such manner, already Ito et al. (1998, p. 887) emphasized that “negative information tends to influence evaluations more strongly than [..] positive information”. Such effect, usually referred to as *negativity bias*, has been detected in several fields of research (Brannon & Gawronski, 2018). Consequently, also Baumeister et al. (2001, p. 323) emphasize the “greater power of bad events over good ones”, concluding that “bad emotions […] and bad feedback have more impact than good ones, and bad information is processed more thoroughly than good”. In such vein, similarly Winkielman et al. (2001, p. 101) conclude in the light of discoveries from brain research “that processing of positive and negative information are not mirror images of each other, but are characterized by different activation functions”. Such empirical findings are also reflected in several renowned conceptual contributions, for instance in the research by Kahneman and Tversky, emphasizing that individuals tend to react much stronger toward negative outcomes from an individual reference point than a comparable positive deviation (e.g., Kahneman, 2012; Kahneman & Tversky, 1979). Such amplified reaction toward negative information is deeply rooted in human evolution and can already be detected in human infants (Hamlin et al., 2010; Vaish et al., 2008), but also in other species (Rozin & Royzman, 2001). Taking these considerations into account, it seems reasonable to assume that the WTI will be more negatively affected by unsustainable behavior than there is a potential increase in WTI given positive sustainability information. Based on this, we come to the following empirical hypothesis:

- **H5** Private investors penalize unsustainable behavior more strongly by reducing their willingness to invest than they reward sustainable behavior by increasing their willingness to invest.

Lastly, also the traditional perspective of shareholder return deserves further scrutiny, especially if one considers situations in which trade-offs between sustainability motives and motives of economic self-interest may occur. In this respect, we know particularly little about the consequences of high-return promises in case of missing corporate sustainability, which is investigated in an empirical research approach. As discussed in our first study, investors certainly consider traditional economic indicators like the return prospects of an investment. However, they even more show a substantial, quite strong concern for the sustainability of businesses. In this context, the question remains whether negative sustainability aspects could be argued to be compensable by higher returns. Although such may be partly the case, our first study has shown the great impact of sustainability issues on WTI, which should also in our second study lead to a generally strong reduction in WTI compared to the control group. Hence, based on these considerations and prior findings, we set forth the following hypothesis:

- **H6** Private investors are less willing to invest into shares of non-sustainable companies with high returns than into companies whose sustainability is not known (control group).

In addition, besides a comparison to the control group, again, a direct comparison of the *non-sustainability high return* to the *non-sustainability average return* group seems essential to estimate the delta between high and low returns for explicit negative sustainability information. Therefore, we set forth our final hypothesis:

- **H7** Private investors are willing to invest more into shares of non-sustainable companies with high returns than into shares of non-sustainable companies with average returns.

### Methods, procedures and sample

Similar to the first study, also the second study is based on the factorial survey approach outlined above. To explore the empirical hypotheses developed for the second study, in total six vignettes were designed. As before, the first vignette represented the control group only asking the participants to place themselves in the scenario of having inherited 10,000 € from which a certain amount could be invested into stocks. The other vignettes added or modified some of this information provided in the control group. The vignettes on positive business sustainability contained additional information on business sustainability commitments, which were furthermore certified by an independent institution. We chose the SA 8000 standard for social and the EMAS certification for environmental sustainability as these are relatively well-known and widely used standards in research and practice regarding the social (Gilbert et al., 2011; Llach et al., 2015) and environmental dimension (Albelda, 2011). Both sustainability standards were furthermore described shortly to ensure that all participants had a similar concept of sustainability in mind when evaluating their investment decision. In such vein, in the second vignette, the participants received additional information on the social commitments of the business, adding that such efforts were certified according to the SA 8000 standard. The third vignette then focused on environmental sustainability, providing the information that the business was environmentally certified in accordance with the EMAS standard. In the fourth vignette, we combined both certification standards to explore whether a combination of sustainability measures could even further increase the individuals’ willingness to invest, compared to the control group as well as the sole provision of only one sustainability certification. Finally, also two vignettes were designed containing an explicit lack of sustainability in business operations. While in the first of these two vignettes, the same information as in the control group was provided concerning the return expectations, the second non-sustainability vignette explicitly promised an increased return of 10–12% to check whether such could compensate for a lack of sustainable behavior (for detailed information see Appendix 1). The design of the vignettes is summarized in Table 2.

**Table 2:** Overview of vignettes

Groups	Vignette	Corporate sustainability information	Certification	Annual return (%)
Control Group	Vc	No information	Not certified	6–7
Treatment Group 1	V1	Socially sustainable	SA 8000	6–7
Treatment Group 2	V2	Environmentally sustainable	EMAS	6–7
Treatment Group 3	V3	Environmentally and socially sustainable	EMAS and SA 8000	6–7
Treatment Group 4	V4	Non-sustainable	Not certified	6–7
Treatment Group 5	V5	Non-sustainable	Not certified	10–12

After designing the vignettes, they were thoroughly pretested by individuals not participating in the study to ensure understandability and precision of the chosen wording. As before, the vignettes were then distributed using a multitude of different channels like university mailing lists and social networks. Correspondingly, also in the second study a between-subjects design was applied to reduce the aforementioned limitations to statistical interpretation if more than one vignette were provided per participant. To ensure that our results would not solely be significant but also possess sufficient statistical power, we conducted an ex-ante power analysis. Assuming a medium effect size of Cohen’s d = 0.5,^4^ we obtained a minimum amount of 51 participants per vignette, thus requiring at least 6 * 51 = 306 participants. In our study, in total 361 participants (187 male, 174 female, else/unspecified: 0) completed the survey with a rather equal distribution of around 60 participants per vignette. As before, the participants did not significantly differ between the vignette conditions regarding socio-demographics or personality traits. Most participants were university students (61.2%), while also several employees participated (24.7%, else: 14.1%). Comparable to the first study, the average age was 27.7 (SD = 8.3) years with a minimum of 14 and a maximum of 73.

### Results

Analyzing our results in more detail, we find that in the control group the participants stated an average willingness to invest of about 4180 €. In the other vignettes, further information was provided on the sustainability of business conduct. Interestingly, when positive information on business sustainability is given, the willingness to invest consistently decreases (descriptively) in comparison to the control group, leaving us with a somewhat puzzling result. While in the environmental sustainability group the willingness to invest drops (− 182.99 €) and even decreases further in the case of social and environmental sustainability (− 187.86 €), the decline in willingness to invest is especially prominent in the case of social sustainability information (− 227.20 €). As already the descriptive deviations are negative, hypotheses H2a-c cannot be empirically supported. Although these decreases are themselves not significant, the fact that all deviations pinpoint to the same direction seems remarkable and might be a fruitful aspect to investigate in further research. In contrast to that, concerning the comparison of combined versus single sustainability contributions, a somewhat different picture emerges. While in the case of social sustainability, at least a descriptive increase can be seen, which is however non-significant (*p* = .469), in the case of environmental sustainability, an additional social commitment already descriptively decreases the willingness to invest. Consequently, also hypotheses H3a and b cannot be empirically supported.

Yet, in the case of non-sustainability in business, a different picture emerges. When given the information of non-sustainable business practices, the participants reduce their willingness to invest by − 1689.83 €, which represents a highly significant (*p* < .001) discount. Therefore, hypothesis H4 is empirically supported by our data. In addition to that, we were also interested in exploring whether the punishment for non-sustainable behavior was more pronounced than the comparable reward due to good sustainable conduct. As in a statistically strict manner, the deviations in case of positive sustainability failed to be significant and therefore must be assumed to be still zero while the negative deviation is highly significant (*p* < .001), also H5 can be supported.

**Fig. 1:**
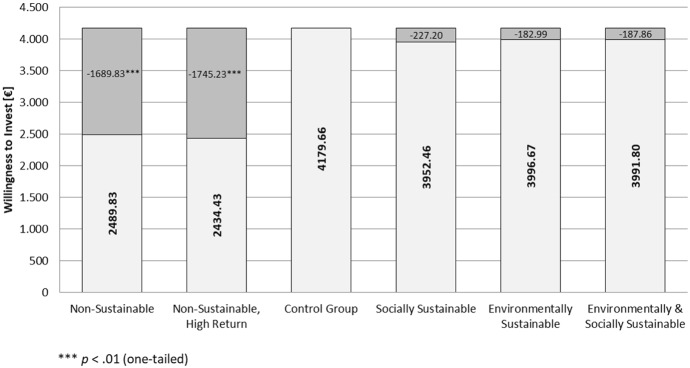
Graphical representation of main empirical results, Study 2

Finally, we investigated whether the incentive of an increased return might exert any influence on an investor’s WTI. Compared to the control group, as before, a highly significant (*p* < .001) reduction in willingness to invest is discernible. Hence, also hypothesis H6 can be empirically supported. Yet, concerning our final hypothesis (H7), interestingly, already descriptively the WTI for non-sustainability and high return is lower than in the comparable standard return group. Thus, H7 is not empirically supported. The major findings of Study 2 are summarized in the following Table 3 and Fig. 1.

**Table 3:** Summary of major empirical results, Study 2

Group	N	Mean [€]	SD [€]	∆ mean sustainability/control group [€]	Sig	∆ mean intergroup comparison [€]	Sig
Control group	59	4179.66	2588.69	–	–	–	–
Socially sustainable	61	3952.46	2912.42	− 227.20	(.326)	− 39.34_a_	.469
Environmentally sustainable	60	3996.67	2835.13	− 182.99	(.357)	4.87_a_	(.496)
Environmentally and socially sustainable	61	3991.80	2584.46	− 187.86	(.346)	–	–
Non-sustainable	59	2489.83	2600.91	− 1689.83	.000***	–	–
Non-sustainable, high return	61	2434.43	2741.22	− 1745.23	.000***	− 55.40_b_	(.455)

t-tests (one-tailed), *p*-values in brackets refer to deviations descriptively in opposite direction of empirical hypothesis. Indices: *a*: comparison with social and environmental sustainability, *b*: comparison with non-sustainable group, ***p < .01

The discovered strong reaction toward non-sustainability in business can also be supported by a descriptive analysis on the distribution of participants explicitly stating that they were not willing to invest into the respective business at all. As can been seen in Table 4, for both non-sustainable average vs. high return there is a substantial amount of participants explicitly stating that they would not invest into the company at all. However, for the high-return group the number of individuals refusing to invest at all is slightly smaller.

**Table 4:** Share of participants indicating an intention of non-investment

	N	No investment (0 €)	
		Absolute [N]	Relative [%]
Total	361	44	12.19
Control group	59	1	1.69
Socially sustainable	61	5	8.20
Environmentally sustainable	60	4	6.67
Environmentally and socially sustainable	61	1	1.64
Non-sustainable	59	18	30.51
Non-sustainable, high return	61	15	24.59

Finally, to further explore and scrutinize our empirical findings, we conducted a series of regression analyses on our vignette scenarios, simultaneously controlling for whether the participants’ socio-demographics, i.e., age, gender, income situation, the status of being student, as well as their investment experience, risk, and sustainability preferences, might have influenced their response behavior to reduce omitted variable bias. In our full model (see Appendix 2, Table 5), we can see that the study’s initial findings are confirmed, as not only the levels of significance are comparable but also the relevant deviations in the treatment groups. Especially reassuring appears to be that as in the first study our in-part student surrogation did not seem to influence the stated willingness to invest. Furthermore, also in the second study most control variables did not exhibit any significant influence. Interestingly however, for risk preferences, there was again a significant positive impact, which seems plausible as less risk aversion corresponds to a greater interest in stock investments. Finally, the participants could also select their investment characteristics most suitable to them, e.g., they could rate themselves as savers, speculative investors, image investors, etc. In this context, most investor types did not show a significant effect. However, savers were significantly less willing to invest into stocks.

In addition (see Appendix 2, Table 6), we further investigated the impact of our control variables. Interestingly, in those partial models, income only appeared to have a weakly significant impact on participants in the control group, while for explicitly sustainable or non-sustainable investments such effect could not be detected. Hence, if no further information on the business is available, it seems that participants are orienting themselves more on their income situation compared to if they can take ethical aspects into their investment consideration. Like in our full model, especially risk preference exhibits an (at least marginally) significant and rather consistent result in several groups, in which a stronger risk preference increases the individual’s willingness to invest. Most striking, however, appears the highly significant impact of sustainability preferences in the non-sustainability, high return group. Therefore, if participants are especially concerned with sustainability matters, they drastically seem to punish non-sustainable behavior if it is linked with high returns.

## Discussion and conclusions

In both studies, we have been exploring the effects of corporate sustainability on an individual’s WTI. After discussing the theoretical background and elaborating the findings and limitations of prior studies, we emphasized the great importance of better understanding the implications of sustainability from a private investor’s perspective. As without sufficient equity, the existence and long-term prosperity of a business is seriously endangered, such investigation is of particular relevance. To explore whether business sustainability might affect an individuals’ willingness to invest, in the first study, we established the sustainability-WTI link. With this study, we are able to show that besides the traditional triad of return, risk, and liquidity also sustainability influences WTI in a substantial manner. In the second study, we consecutively explored this relationship in greater detail. Here, we find that the link between sustainability and WTI is asymmetric. Hence, while exceeding the general expectations of good business practice might not additionally increase WTI, violating general norms of good corporate conduct leads to a significantly reduced WTI, which cannot even be compensated by increasing return prospects. Therefore, in the context of sustainability and WTI, it seems less an issue whether it may “pay to be good” but rather whether it “harms to be bad”, indicating that especially the latter might be highly interesting for future studies.

Consequently, our paper contributes to present research in several ways. At first, it establishes a clear indication that the traditional triad of investment is indeed not sufficient, demonstrating a strong link between sustainability and WTI on an empirical basis. Furthermore, we can show that the impact of sustainability is asymmetric; therefore, positive and negative conduct of businesses cannot be treated alike. While increasing sustainability efforts are not rewarded, a clear lack of sustainability is significantly punished. These findings also have several implications for Management Accounting and the design of Management Control systems. At first, our studies show that “sustainability matters”, i.e., we can substantiate the relevance for increasingly measuring corporate sustainability performance and integrating these indicators in internal as well as external reports. In addition, our second study explicitly differentiates between positive and negative deviations in sustainability performance. For Management Accounting, our findings imply that besides a general measurement of sustainability performance a greater emphasis could be placed on discussing and evaluating existing “minimal thresholds” for sustainability, which corporate conduct should not overstep. In such vein, management could be advised to take countermeasures if specific sustainability indicators are getting dangerously low (“red flags”). Besides supporting management and stakeholders with relevant information, our findings also deliver some implications for Management Accounting concerning the design and implementation of corporate incentive schemes. In this context, our empirical results show once more the great relevance to think about incentive schemes that support maintaining a sufficiently sustainable business conduct and specifically ensuring that non-sustainable behavior is not encouraged.

For future research, building upon the results of our two studies, several highly relevant aspects remain to be investigated. At first, it would be beneficial to better understand why on average sustainability commitments did not increase an investor’s WTI. One possible answer to explain the non-significance of positive sustainability efforts could be found in the so called ELSI (ethical = less strong intuition) theory, stating that ethical goods are often thought to be less performant than their regular counterparts (Mai et al., 2019), although research shows a much more nuanced picture (Kim, 2017; Revelli & Viviani, 2015). Still, individuals could consistently assume that a particularly ethically motivated company would not economically perform as well as a standard business. Furthermore, from a perspective of schematic fit, we also hypothesized that a combination of sustainability measures could further increase WTI in comparison to only one-dimensional social or environmental sustainability commitments. However, even with combined efforts, more sustainability did not lead to a significant increase in WTI. Rather, as our results show, the WTI of combined sustainability measures is quite similar to single sustainability commitments by the respective business. Why this is the case would be relevant to investigate in future research. One explanation could be that already with the information on only one sustainability dimension the business is classified as a sustainable business, which does not systematically change, even when the sustainability efforts are complemented by further sustainability measures.

Additionally, also on the side of reduced WTI due to a lack in sustainability it would be interesting to further explore the motives for such reduction. While on the one hand, the reason for conceiving a non-sustainable business unit as less attractive might be based on true ethical concerns, on the other hand also motives of prudence (for a discussion see Arnold et al., 2013) could be influential as such business units may face legal claims or other turmoil by stakeholders’ withdrawal of resources. Hence, it seems interesting for upcoming research to shed more light on the reasons for a decrease in willingness to invest in cases of non-sustainability.

Yet, that besides motives of self-interest also genuine ethical concerns are relevant is suggested by the findings of the second study, showing that even an increased return cannot easily compensate overstepping good corporate conduct. If one follows such considerations, the persistent neglect of widely held expectations of minimal business ethical standards might have much further reaching consequences. Such conduct might in the end seriously undermine the legitimacy of the business unit, damaging the general societal perception of the rightfulness and appropriateness of business practices, which finally may lead to a withdrawal of the societally granted “license to operate” (for such concept see e.g., Morrison, 2014; Reinhardt, 2005; Thomson & Boutilier, 2011; Wilburn & Wilburn, 2011). As several scandals from the past demonstrate, losing stakeholders’ perception of legitimate corporate conduct might seriously threaten the long-term existence of a business when investors intend to sell their shares, or short-term oriented, opportunistic traders are finally the only investors remaining. In addition, it might also become seriously difficult to sell the business’s products and services or to acquire talented and motivated individuals for employment. Yet, and quite interestingly, in the case of non-sustainability, our data hints to the possibility that an increased return might to some degree at least prevent some individuals from not investing at all, also deserving more research in future studies.

Furthermore, the present studies focused on private investors. Consequently, it might be interesting to explore whether future research could confirm our results in the context of institutional investors. In addition, there exist already several conceptual as well as empirical studies focusing on the character traits of investors (e.g., Anand & Cowton, 1993; Beal et al., 2005; Hudson, 2005; Webley et al., 2001). As our extended results show, in the vignette of non-sustainability with increased returns, especially individuals with strong sustainability preferences significantly punished unethical behavior. Therefore, it seems of high interest to use such character traits as moderators on the sustainability-WTI link in future studies. In such line of thought, one might consequently assume that individuals with dark character traits like corporate psychopathy (e.g., Paulhus & Williams, 2002) could be expected to be totally unaffected by sustainability issues as they are anti-social and risk-seeking (Babiak, 1995; Babiak & Hare, 2007; Boddy, 2011; Boddy et al., 2010).

For future studies, it also seems of interest to investigate a setting in which participants would not solely invest but also be provided with the possibility to withdraw their investment, which appears especially interesting to explore in the case of non-sustainability. Connecting to this, one could also vary the participants’ response opportunities in investigating not only a possible withdrawal from or (an additional) investment into a certain business but also the holding period (Paetzold & Busch, 2014), which could be connected with the investor traits as a moderating variable. In addition, both studies chose a sum of 10,000 € to potentially invest into company stocks. We chose such value as for the average participant it should represent a tangible sum, enhancing the immersion and realism of the psychological environment, relevant for obtaining realistic responses (Aguinis & Bradley, 2014). In the future, it would be interesting to vary such available sum and furthermore take into account the prior endowment of participants, as with increasing resources available, more money might be invested as it is not needed for daily life consumption (Eurosif, 2012). Also, future studies could investigate deeper into the link between trust in certification and sustainability, which equally could be applied as a moderating variable. For several of these highly relevant research questions the utilized methodology of the factorial survey seems promising. Finally, our studies relied on the vignette methodology in an online survey. For future studies, it could be interesting to apply other methods like laboratory experiments, where for instance, participants could invest some of their own money in different scenarios. Insights from these experiments could then be coupled with field data to substantiate our findings, which appears particularly interesting from a perspective of method triangulation (Campbell & Fiske, 1959; Denzin, 2009; Patton, 1999).

Besides these open research questions and as a first cautious interpretation of the results presented, we think that both studies show that alongside traditional economic performance indicators it is essential for business to ensure that corporate conduct stays within the boundaries of widely held normative assumptions and values in society. Above-average sustainable behavior therefore need not always pay off in terms of increasing investment attractiveness but there are strong indications that persistent violations of moral standards are harming WTI, if not taken care of. Our findings in this paper therefore seem a further step in complementing the rich and fruitful research in ethical decision-making, explicitly focusing on an investor’s perspective.

## Data Availability

Available upon request.

## Appendix 1: Vignettes Study 1 and 2^5^

### Study 1

General Information (all vignettes):

Please put yourself in the following situation:

You have recently inherited 10,000 €, which you can spend, for example, on an equity investment.

In doing so, you came across shares in a company about which you have the following information from the last few years. You can assume that these assumptions will also apply to the coming years, taking into account the overall economic situation:

Factor *Return* (good/bad outcome):

The stock has achieved a return of 10–12% per year, which is well above the market average./The stock has achieved a return of 3–4% per year, which is well below the market average.

Factor *Risk* (good/bad outcome):

The return was subject to lower fluctuations than those usual on the stock market. In addition, there is a consensus among the rating agencies that it is a safe investment./The return was subject to greater fluctuations than those usual on the stock market. In addition, there is a consensus among the rating agencies that this is a speculative investment.

Factor *Liquidity* (good/bad outcome):

The share is traded on a stock exchange and you can assume that you can sell it at any time due to the high demand./The share is traded on a stock exchange and you can assume that you may not be able to sell it at any time due to limited demand.

Factor *Sustainability* (good/bad outcome):

The company has the EMAS seal and the SA 8000 standard, which certify ecologically and socially sustainable behavior, and is also actively involved in social and ecological projects. (EMAS & SA 8000 logo)/The company does not have any seals or certificates that certify ecologically or socially sustainable behavior and is also repeatedly confronted with accusations of corruption and has been involved in several environmental scandals in recent years.

### Study 2

Control Group:

Please put yourself in the following situation:

You have recently inherited 10,000 €, which you can spend, for example, on an equity investment.

You have come across shares of a company that have achieved a market average return of 6–7% per year in recent years, with the usual fluctuations on the stock market. You can assume that similar annual returns will be achieved on the company’s shares over the next few years, taking into account market fluctuations.

Socially Sustainable:

[Information Control Group].

The company is SA 8000 certified. This certifies socially sustainable behavior such as the renunciation of child and forced labor, compliance with regulated working hours and minimum standards in the areas of health protection and occupational safety.

(SA 8000 logo).

Environmentally Sustainable:

[Information Control Group].

The company holds the EMAS seal of approval. This certifies environmentally sustainable behavior in the areas of energy consumption, emissions or biological diversity.

(EMAS logo).

Environmentally and Socially Sustainable:

[Information Control Group].

The company holds the EMAS seal of approval. This certifies environmentally sustainable behavior in the areas of energy consumption, emissions or biological diversity. In addition, the company has been certified with the SA 8000 standard, which demonstrates socially sustainable behavior such as the renunciation of child and forced labor, compliance with regulated working hours and minimum standards in the areas of health protection and occupational safety.

(EMAS and SA 8000 logo)

Non-Sustainable:

[Information Control Group].

The business is repeatedly confronted with allegations of corruption and has been involved in several environmental scandals in recent years.

Non-Sustainable, Increased Return:

[Introduction Control Group].

You have come across shares of a company that have achieved a market average return of 10–12% per year in recent years, with the usual fluctuations on the stock market. You can assume that similar annual returns will be achieved on the company’s shares over the next few years, taking into account market fluctuations.

The business is repeatedly confronted with allegations of corruption and has been involved in several environmental scandals in recent years.

## Appendix 2: Regression Analyses Study 2

See Tables 5 and 6.

**Table 5:** OLS regression results: full model

	Beta	SE	t	Sig
(Constant)	4894.183	2283.864	2.143	.033
Social sustainability	185.941	517.785	.359	.720
Environmental sustainability	63.524	509.137	.125	.901
Environmental and social sustainability	133.353	518.572	.257	.797
Non-sustainability	− 1226.732	514.972	− 2.382	.018**
Non-sustainability and High return	− 1254.949	515.730	− 2.433	.016**
*Control variables*				
Age	− 28.708	20.159	− 1.424	.155
Gender	− 46.771	332.743	− .141	.888
Income	216.883	168.243	1.289	.198
Student	− 67.650	491.342	− .138	.891
Investment experience	− 109.032	168.531	− .647	.518
Risk preference	607.766	167.678	3.625	.000***
Sustainability preference	− 20.252	156.824	− .129	.897
Self-assessment: savers	− 1158.548	474.326	− 2.443	.015**
Self-assessment: speculators	− 16.518	454.698	− .036	.971
Self-assessment: image investor	877.470	616.116	1.424	.155
Self-assessment: critics	58.979	483.023	.122	.903
Self-assessment: employee shareholders	201.257	633.798	.318	.751
Self-assessment: business angels	− 596.761	733.941	− .813	.417
Self-assessment: ethical investors	− 461.858	498.174	− .927	.355

R^2^ = .183. N = 330, 31 participants omitted as they did not fully answer all items

Gender coding: male = 0, female = 1. Beta: Unstandardized regression coefficients

Dependent variable: Willingness to Invest. ***p* < .05, ****p* < .01

**Table 6:** OLS regression results: controls, specific vignettes

	Control group		Social		Environmental		Environmental and social		Non-sustainable		Non-sustainable, high return	
	Beta	Sig	Beta	Sig	Beta	Sig	Beta	Sig	Beta	Sig	Beta	Sig
(Constant)	9336.610	.298	7836.048	.167	3606.892	.578	7048.179	.140	− 2809.034	.645	1002.370	.882
Age	− 4.489	.961	− 8.693	.875	− 51.232	.242	− 39.481	.468	− 13.688	.775	34.233	.575
Gender	190.105	.824	− 954.308	.313	752.379	.392	817.485	.354	1243.208	.158	− 430.395	.651
Income	932.631	.069*	− 282.674	.600	21.341	.966	− 76.452	.814	458.465	.305	− 284.330	.550
Student	850.246	.578	− 95.850	.946	− 234.713	.859	− 751.587	.457	1036.566	.479	− 952.100	.474
Investment Experience	− 199.209	.684	− 516.231	.223	− 126.377	.790	763.993	.082*	34.731	.939	− 430.542	.328
Risk preference	618.974	.192	880.164	.031**	659.829	.249	683.861	.072*	710.409	.163	− 73.819	.872
Sustainability preference	255.450	.495	667.393	.078*	97.092	.847	− 97.479	.821	713.798	.132	− 1395.990	.002***
Savers	− 2424.355	.111	− 1885.595	.120	1132.864	.455	− 1802.242	.105	− 1518.587	.338	− 160.065	.902
Speculators	− 1941.273	.174	1526.411	.191	1322.214	.319	− 1305.604	.223	187.598	.862	527.336	.684
Image investor	− 505.874	.880	508.438	.774	1069.794	.521	− 759.574	.559	1556.261	.374	− 322.096	.862
Critics	− .812	1.000	− 1769.447	.196	− 771.680	.664	645.499	.536	869.855	.480	1582.010	.324
Employee shareholders	207.412	.915	1936.500	.421	− 2581.793	.111	(n.a.)^+^	(n.a.)^+^	2919.352	.063**	1728.454	.274
Business angels	− 3253.582	.274	(n.a.)^+^	(n.a.)^+^	221.740	.900	− 994.594	.446	− 2289.230	.276	86.643	.961
Ethical investors	626.294	.613	− 1998.985	.222	− 1257.104	.442	340.290	.848	411.383	.712	− 388.461	.818
Model R^2^	.393		.427		.188		.357		.335		.392	
N	50		54		58		57		56		55	

Total N = 330, 31 participants omitted as they did not fully answer to all items. Gender coding: male = 0, female = 1. Beta: Unstandardized regression coefficients. Dependent variable: Willingness to Invest, ^+^excl. from regression since item not chosen in resp. dataset. **p* < .10; ***p* < .05, ****p* < .01

## Footnotes

1 Some institutions like Eurosif (2016) and some authors in SRI literature like Sjöström (2011), Revelli and Viviani (2015), or Renneboog et al. (2008a) define SRI broader as an investment process that combines the investor’s financial objectives with concerns about environmental, social, and corporate governance (ESG) issues known as ESG factors.

2 The issue of screening is extensively discussed in SRI literature (see for example Barnett & Salomon (2006), Von Wallis & Klein (2015), and Trinks & Scholtens (2017)). In this context, some authors like Schwartz (2003) argue that for most “questionable” industries, negative as well as positive effects can be found.

3 Further assumptions: alpha = .05, power = .80.

4 Further assumptions: alpha = .05, power = .80.
